# Cholesterol-Lowering and Liver-Protective Effects of Cooked and Germinated Mung Beans (*Vigna radiata* L.)

**DOI:** 10.3390/nu10070821

**Published:** 2018-06-26

**Authors:** Lays Arnaud Rosal Lopes, Maria do Carmo de Carvalho e Martins, Luciana Melo de Farias, Ana Karolinne da Silva Brito, Geovanni de Morais Lima, Vanessa Brito Lira de Carvalho, Cristian Francisco de Carvalho Pereira, Aírton Mendes Conde Júnior, Tatiana Saldanha, José Alfredo Gomes Arêas, Kaesel Jackson Damasceno e Silva, Karoline de Macêdo Gonçalves Frota

**Affiliations:** 1 Department of Nutrition, Universidade Federal do Piauí, Campus Universitário Ministro Petrônio Portela, Teresina, PI 64.049-550, Brasil; lays_rosal@hotmail.com (L.A.R.L.); lmdefarias@yahoo.com.br (L.M.d.F.); vanessablcarvalho@gmail.com (V.B.L.d.C.); 2 Department of Biophysics and Physiology, Universidade Federal do Piauí, Campus Universitário Ministro Petrônio Portela, Teresina, PI 64.049-550, Brasil; carminhamartins@yahoo.com.br; 3 Department of Pharmacology, Universidade Federal do Piauí, Campus Universitário Ministro Petrônio Portela, Teresina, PI 64.049-550, Brasil; anakarolinnesb@hotmail.com (A.K.d.S.B.); moraisgeovanni@gmail.com (G.d.M.L.); 4 Department of Anatomy and Morphology, Universidade Federal do Piauí, Campus Universitário Ministro Petrônio Portela, Teresina, PI 64.049-550, Brasil; cristian_cfcp@hotmail.com (C.F.d.C.P.); airton.conde@ufpi.edu.br (A.M.C.J.); 5 Department of Food Technology, Universidade Federal Rural do Rio de Janeiro, Rodovia BR 465, Km 07, s/n—Zona Rural, Seropédica, RJ 23890-000, Brazil; tatysal@gmail.com; 6 Faculty of Public Health, Department of Nutrition, Universidade de São Paulo, Av. Dr. Arnaldo, n. 715, São Paulo, SP 12.406-904, Brasil; jagareas@usp.br; 7 Empresa Brasileira de Pesquisa Agropecuária—EMBRAPA, Av. Duque de Caxias, nº 5.650, Bairro Buenos Aires, Teresina, PI 64008-780, Brasil; kaesel.damasceno@embrapa.br

**Keywords:** protein, vigna, germination, lipid metabolism, fatty liver, cardiovascular disease

## Abstract

We investigated the hypocholesterolemic and liver-protective effects of cooked and germinated whole mung beans. Hamsters were fed for 28 days on diets rich in saturated fatty acids and cholesterol, differing only in protein source (20%): casein, cooked whole mung bean, and germinated mung bean. After 28 days, we found reduced plasma concentrations of total cholesterol and non-HDL cholesterol, increased faecal cholesterol excretion, and reduced levels of asparagine aminotransferase and alanine aminotransferase enzymes in the liver. Reduction in hepatic lipid deposition was observed between each of the mung bean groups relative to the casein group. In addition, the animals of the geminated mung bean group showed a lack of inflammatory infiltrate and better vascularisation of the hepatic tissue. Results from this study show significant hypocholesterolemic and liver-protective properties of the mung bean, which are further enhanced after germination.

## 1. Introduction

Elevated plasma cholesterol is a major risk factor for cardiovascular disease (CVD), causing 2.6 million deaths world-wide each year [1,2]. A high intake of cholesterol, simple carbohydrates, saturated fatty acids, and *trans* fatty acids, in addition to excessive calorie consumption, are related to increased serum cholesterol and triacylglycerol levels [3].

This imbalanced dietary intake contributes to the excessive accumulation of triacylglycerols in hepatocytes, which characterises non-alcoholic fatty liver disease (NAFLD). The excess of triacylglycerols in hepatocytes can reach toxic levels, which generate increased oxidative stress with the formation of free radicals, associated with mitochondrial dysfunction. Abnormal lipid peroxidation results in direct hepatic damage, with inflammation and even fibrosis [4,5].

Dietary interventions with legumes aimed at reducing cardiovascular diseases have received special attention around the world because of the hypocholesterolemic and liver-protective properties of legumes. Modulation of plasma cholesterol levels by the consumption of soybean and other legumes, such as lupine, chickpeas, lentils, and beans, has been demonstrated in animal and human studies. In addition, reduction in hepatic lipid deposition and prevention of the occurrence of steatosis have been reported in animal intervention experiments [6,7,8,9,10,11].

Previous studies have demonstrated that several components occurring in legumes could be responsible for such effects, such as bioactive compounds and dietary fibre, as well as constituent proteins due to their composition of specific amino acids and bioactive peptides [12,13,14].

The mung bean (*Vigna radiata* L.) is a legume produced in China, Burma, India, Korea, Pakistan, Japan, Thailand, and other parts of Southeast Asia [15]. Its cultivation in the West has increased due to a growth in demand for the germinated grain (bean sprout), the form in which it is mostly consumed [16]. The germination process involved in bean sprout production is simple and inexpensive. From a nutritional point of view, germination of the mung bean increases its protein content and digestibility, promotes an increment in the content of bioactive compounds, and reduces anti-nutritional factors [15,17,18,19,20]. However, to date, no studies have been published on the effect of germinated mung beans in the diet on plasma cholesterol levels, and there are few animal studies that have investigated whole mung beans [21,22,23]. 

It is known that the main components of leguminous seeds which respond for cholesterol-lowering and liver-protective effects are proteins and bioactive peptides, with the latter formed during seed digestion [7,8,13,14,24]. The germination process increases the protein content and, consequently, the peptides in the gut during legume protein digestion. It is also well established that hamsters are the most appropriate animal model for dietary intervention studies. Hamsters, similar to humans, are hyper-responsive and have similar plasma ratios of High-Density Lipoprotein cholesterol (HDL-cholesterol) and non-HDL-cholesterol in plasma, in addition to having the same cholesterol transport mechanism [25]. Therefore, the objective of this study was to investigate the hypocholesterolemic and liver-protective effects of cooked mung beans and sprouted mung beans in hamsters with dietary-induced, elevated cholesterol levels.

## 2. Materials and Methods

### 2.1. Preparation of Samples

Mung beans (*Vigna radiata* L.), cultivar MGS-Esmeralda, were supplied by Embrapa Meio-Norte, Teresina, PI, Brazil. The bean seeds were sterilised with sodium hypochlorite solution at 300 ppm for 10 min and washed with plenty of water. 

Cooked mung beans were prepared in an autoclave (Prismatec, model CS-30) at 120 °C for 15 min (bean:water ratio 1:2 w/v). The seed germination methodology was adapted from Huang, Cai, & Xu [26]. The washed beans were soaked for 10 h in water at room temperature (30 °C). Water was then drained off and beans were placed in sterilised polyethylene containers, covered with cotton and with holes at the bottom to allow the drainage of water. The containers were placed in a germination chamber (Marconi, model MA-401) for 72 h, in the absence of light, with circulating air, 100% relative humidity, and temperatures controlled in the range of 25 °C to 35 °C. During the germination period, the shoots were sprayed daily with drinking water.

Cooked mung beans and germinated mung beans were dried in a ventilated oven at 50 °C for 72 h, milled in an analytical mill (IKA, model A11), and sieved (35 mesh), yielding two different flours: Whole cooked Mung Bean Flour (WMBF) and Germinated Mung Bean Flour (GMBF).

### 2.2. Animals, Diets and Experimental Procedure

This research protocol was submitted to the Ethics Committee on Animal Experimentation of the Federal University of Piauí and approved under protocol number 01.0264.2014. All procedures related to animal handling were carried out according to recommendations in the “Guide for the Care and Use of Laboratory Animals” [27]. A total of 37 male hamsters, recently weaned with the conventional sanitary standard, were obtained from Anilab Animais de Laboratório, Criação e Comércio LTDA—EPP, Brazil. The animals were housed in individual cages in a ventilated environment, with temperatures of approximately 20 °C to 25 °C, 55% relative humidity, exposed to a light-dark cycle of 12 h, and fed ad libitum.

Preliminary tests were performed in a pilot study using five animals, to ensure that the induction of hypercholesterolemia would be achieved by feeding a Casein Hypercholesterolemic Diet (CHD), rich in saturated fatty acids (13.5%) and cholesterol (0.1%), for a period of 21 d. After confirmation of the elevation of all lipid parameters, evaluated before and after the induction period, the main experiment was carried out.

For the main experiment, all the animals underwent a 20-day acclimatization period during which they received a Commercial Diet (CD) with the aim of reducing stress caused by their transfer. After this period, eight animals were randomly separated and continued to receive CD until the end of the experiment, representing the Negative Control Group (NCG) (*n* = 8). The remaining animals received CHD for a period of 21 days to induce hypercholesterolemia.

After hypercholesterolemia had been achieved, hamsters were randomly divided among three groups, each containing eight animals. Each group received a different diet over a period of 28 days. One group continued with CHD and was termed the positive control group (PCG) (*n* = 8). A second group received the Whole cooked Mung Bean flour Diet (WMBD), an experimental diet containing WMBF, as well as the addition of saturated fatty acids (13.5%) and cholesterol (0.1%), and was denominated the Whole cooked Mung Bean flour Group (WMBG) (*n* = 8). The third group received the Germinated Mung Bean Diet (GMBD), an experimental diet containing GMBF, as well as the addition of saturated fatty acids (13.5%) and cholesterol (0.1%), and was denominated the Germinated Mung Bean Flour group (GMBG) (*n* = 8). At the end of the 28-day experimental period, blood samples were collected from all animals for biochemical analysis.

The calculations for the different components of the experimental diets containing cooked or germinated mung beans were based on the centesimal analysis performed by official methods [28], as shown in Table 1. Therefore, the formulations of the diets containing whole cooked and the germinated bean flours resulted in diets with the same protein, lipid, and calorie contents, also providing the same amounts of cholesterol and choline chloride, and differing only in the protein source. The commercial diet was formulated by the manufacturer. Diets containing WMBF and GMBF were supplemented with L-methionine, an amino acid which is typically low in diets containing legume protein [29].

Details of the formulation of the control and experimental diets, as well as the centesimal composition performed by official methods [28], are shown in Table 2.

### 2.3. Parameters of Growth and Food Consumption

During the experiment, the animals were weighed twice weekly to determine weight gain. Feed was weighed and changed on a daily basis in order to determine the amount consumed by each animal, excluding any leftover feed. Food Efficiency Ratios (FER) were determined for each diet based on the ratio of weight gain and amount of diet consumed during the 28-day experimental diet period.

### 2.4. Sample Collection

During the last five days of the experiment (day 24 to 28), animal faeces were collected, oven-dried at 45 °C for 36 h, and ground for subsequent analysis.

To perform the blood collection procedure, animals were subjected to general anaesthesia by the initial application of lidocaine (10 mg/mL), followed by thiopental sodium at a dose of 100 mg/kg body weight by intraperitoneal injection. Blood samples were then collected from the vena cava. All blood collections took place after a fasting period of 10 to 12 h.

Blood samples were transferred into tubes containing heparin anticoagulant (Liquemine) at a final concentration of approximately 1 mg/mL. After a period of a mimimum of one hour (maximum of two hours), blood samples were subjected to low speed centrifugation (10,000 rpm, 15 min, 4 °C) to obtain plasma for subsequent analysis.

After blood collection, animals were sacrificed by exsanguination. Livers were then removed, weighed, and preserved in 10% buffered formalin for 48 h for subsequent histological examination.

### 2.5. Plasma Lipid and Lipoprotein Analyses

Plasma total cholesterol and triacylglycerol concentrations were measured using commercial enzyme assay kits (Labtest, Lagoa Santa, Brazil). HDL-cholesterol was measured after the precipitation of apoB-containing lipoproteins using sodium phosphotungstate magnesium chloride (Labtest, Lagoa Santa, Brazil). Total cholesterol in the supernatant fraction was determined using an enzyme kit, in accordance with Weingand and Daggy [30]. The cholesterol concentration in the Very Low-Density Lipoprotein (VLDL) + Low-Density Lipoprotein (LDL) fraction was expressed as non-HDL cholesterol and was calculated as the difference between total plasma cholesterol and HDL cholesterol. Although hamsters have a lipoprotein profile similar to humans, the distribution of triacylglycerol across the lipoprotein groups differs to that of humans [31]. Consequently, the calculation of LDL-cholesterol using the equation of Friedewald, Levy and Fredrickson [32] is inappropriate.

### 2.6. Analysis of Faecal Cholesterol

Cholesterol concentrations in oven-dried faeces were analysed after performing cholesterol extraction using hexane. The residue was dissolved in 5 mL of hexane, transferred to a screw top flask, dried under nitrogen, diluted with 1 mL of mobile phase, filtered through a 22 µm membrane (Millipore, Burlington, MA, USA), and injected in the HPLC system, according to Saldanha et al. [33]. For HPLC, Waters liquid chromatography (Waters, Milford, MA, USA) was used, equipped with on-line PDA (Waters 2998) and refractive index (RID-Waters, 2414) detectors, a rheodyne injector with a 20 µL loop, a tertiary solvent delivery system (Waters 600), an oven heated column at 32 °C ± 1 °C (CTO-3840), and software (Empower 2). The analytical column was a Nova Pack CN HP 300 mm, 3.9 mm, 4 mm (Waters, Milford, MA, USA). The mobile-phase was n-hexane: 2-propanol (97:3, v/v) at a flow rate of 1 mL/min and analysis time of 30 min. The HPLC solvents were filtered through a 22 mm Millipore membrane (Bedford, MA, USA) under vacuum prior to use. Quantification was done by external standardization, with a concentration range from 0.1 to 1.8 mg/mL.

### 2.7. Liver Function and Liver Damage

Plasma concentrations of total proteins and albumin were measured as indicators of liver function, and plasma concentrations of aspartate aminotransferase (AST) and alanine aminotransferase (ALT) were determined as indicators of liver damage. Analysis was conducted using commercial enzyme assay kits (Labtest, Lagoa Santa, Brazil).

### 2.8. Histological Evaluation

After the sacrifice of animals, livers were removed and preserved in 10% buffered formalin for at least 48 h. Three cross-sectional samples were taken from each liver, dehydrated, and impregnated in paraffin wax. Sections were cut at a thickness of 5-μm, mounted on glass slides, and subsequently stained with haematoxylin-eosin. Histological sections were examined under a light microscope (Olympus, Tokyo, Japan) and photomicrographs prepared using a digital photomicrographic system (Nikon Eclipse E200, Tokyo, Japan). Analysis of histological sections was performed using image analysis software (Belmicro image analyser).

Histological evaluation of the liver included semi-quantitative analysis of the histopathological findings. For this analysis, all the slides were coded and analysed by a pathologist without previous knowledge of animal treatments. Histopathological changes were graded on a scale of 1+ to 4+, where: 

1+ = normal, healthy liver tissue (no changes); 

2+ = liver tissue with a slight increase in the hepatocyte nucleus/cytoplasm ratio, mild capillary congestion; absence of steatosis (mild changes);

3+ = liver tissue with a moderate increase in the hepatocyte nucleus/cytoplasm ratio, mild vascular congestion, increased cellularity, and presence of mild inflammatory infiltrate; focal steatosis (moderate changes); 

4+ = liver tissue with loss of hepatocyte cell delimitation, vacuolar degeneration, severe inflammatory infiltrate, large numbers of macrophages and Kupffer cells in areas of congestion, and necrosis; extensive steatosis.

### 2.9. Statistical Analysis

The results were expressed as the mean and standard error. For a comparison of mean values, the Kolmogorov-Smirnov test was first applied to evaluate the normality of the data. When the data presented a normal distribution, an analysis of variance (ANOVA) was used to compare mean values. When there was a significant difference, Tukey’s-HSD multiple comparison test was applied at a significance level of 5%. For data with a non-normal distribution, the Kruskal-Wallis test was used; when there was a statistical difference, the non-parametric Mann-Whitney test was used, at a significance level of 5%. Statistical analysis was conducted and graphs constructed using SPSS 10.0 (USA) software.

## 3. Results and Discussion

The chemical compositions of whole cooked mung bean flour and germinated mung bean flour on a dry matter basis are shown in Table 1. The results show a higher protein content, as well as a lower carbohydrate and lipid content, in germinated mung beans compared to whole cooked mung beans. Planned composition and centesimal analyses of the diets are presented in Table 2. Formulation of the two experimental diets was based on the chemical composition of the whole cooked mung bean flour and germinated bean flour (Table 1). These diets and the casein hypercholesterolemic diet (CHD) presented a similar composition with the same calorie, protein, carbohydrate, and lipid content, with the exception of the protein source. Protein content of the commercial diet was similar to the other diets; however, lipid and calorie content were lower.

**Figure 1:**
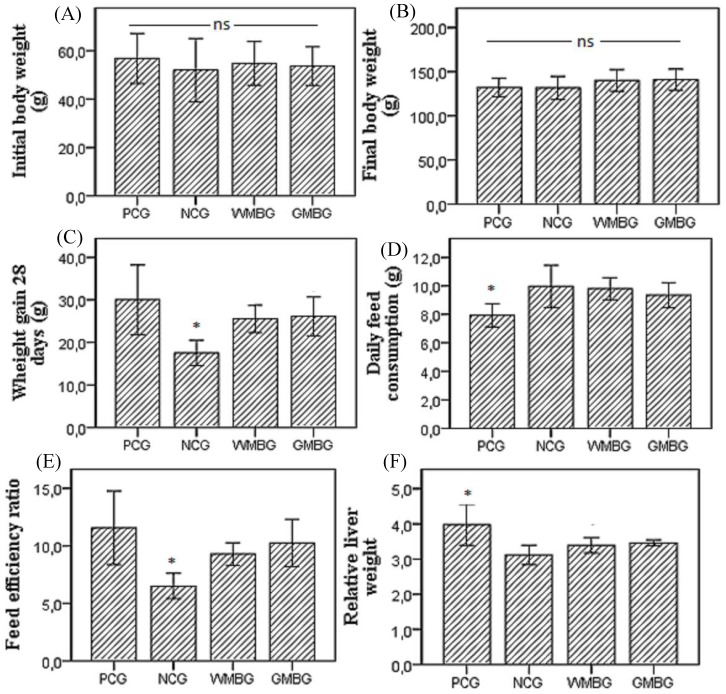
Initial body weight (**A**); Final body weight (**B**); Weight gain 28 days (**C**); Daily feed consumption (**D**); Feed efficiency ratio—FER (**E**); Relative liver weight to body (**F**) in positive control (PCG), negative control (NCG), and experimental groups fed diets containing whole (cooked) mung bean flour (WMBG) and germinated mung bean flour (GMBG). (FER = weight gain/diet ingested × 100 g/100 g body weight); Mean ± SD. * statistically different at *p* < 0.05 (ANOVA followed Tukey’s test), (ns = no significant difference).

The parameters of growth, feed intake, and liver weight of the animals are shown in Figure 1.

The initial body weight and the final body weight of the animals showed no significant difference between the groups. Weight gain during the 28 days of the experiment and the FER of the Negative Control Group (NCG) was lower than the other groups, which is explained by the fact that this group of animals did not receive hypercholesterolemic agents in the diet. Although daily food consumption in PCG animals was lower than in other groups, weight gain in the 28 days of the experiment or FER did not differ compared to WMBG and GMBG animals.

Relative liver weight (g/100 g BW) in the PCG animals was significantly greater than in the other groups; there was no significant difference in relative liver weight between NCG, WMBG, and GMBG animals. The higher relative liver weight reported in PCG animals is related to a higher accumulation of cholesterol and fatty acids in the liver. Liao, Lin, & Kuo [34] observed a significantly higher triacylglycerol and total cholesterol content in the liver of hypercholesterolemic hamsters, and a higher weight of these lipids relative to liver weight in hypercholesterolemic animals compared to non-hypercholesterolemic animals.

Plasma lipid concentrations and faecal excretion of cholesterol of the animals at the end of the experiment are shown in Table 3.

There was no significant difference between WMBG and GMBG in the lipid profile parameters evaluated. The two groups produced similar results, with a significant reduction in plasma concentrations of non-HDL cholesterol (non-HDL-c) compared to PCG, so that both cooked and germinated beans showed a hypocholesterolemic effect. There was no significant difference in concentrations of triacylglycerols (TG) or HDL-cholesterol (HDL-c) compared to PCG.

The hypocholesterolemic effect of mung beans has been previously reported by Yao et al. [22]. These authors reported a significant reduction in plasma concentrations of Total Cholesterol (TC), non-HDL-c, and TG in hamsters with the whole cooked mung bean addition to the diet. Other legumes have also showed hypocholesterolemic effects. Fontanari et al. [7] and Frota et al. [8], using lupine and cowpea, respectively, showed a significant reduction in TC and non-HDL-c concentrations, even in the presence of excess fatty acids and cholesterol. Studies indicate that legume proteins, and peptides derived from them during digestion, act to reduce plasma concentrations of cholesterol. Several mechanisms are involved, including the regulation of activities of key enzymes involved in cholesterol metabolism, such as fatty acid synthetase, 3-hydroxy-3-methyl-glutaryl-CoA reductase (HMG-CoA), HMG-CoA synthetase, and cholesterol 7-α-hydroxylase [13,22,24,35].

In addition, genetic mechanisms appear to be involved, such as the reduction in gene expression of mRNA of sterol regulatory-element binding protein1 (SREBP-1), a transcription factor regulating the expression of genes related to fatty acids. A reduction in mRNA expression of the enzyme fatty acid synthase (FAS) and Apo-B mRNA has been observed [36], as well as a reduction in the gene expression of SREBP-1 mRNA in diet-induced hypercholesterolemic rats treated with mung bean protein isolate [21].

There was a higher faecal excretion of cholesterol in WMBG and GMBG animals compared to PCG animals; this increase probably contributed to the reduction in plasma concentrations of total cholesterol and non-HDL cholesterol.

In vivo studies have shown that proteins from other legumes, such as soybean, cowpea, and lupine, promote increased faecal excretion of cholesterol and bile acids, and that whole grains promote even greater excretion [7,8,37].

The increased faecal excretion of cholesterol promoted by legume proteins is related to inhibition of the micellar solubilisation of cholesterol, a process that is essential for the absorption of cholesterol, by peptide fractions of these proteins, due to the presence of sequences of hydrophobic peptides that compete with cholesterol and bile acids in the micellar organisation [24]. 

In addition to proteins, bioactive fibre and compounds such as phenolic acids present in legumes are also noted for their ability to form complexes with cholesterol, acids, and bile salts, decreasing their intestinal absorption and increasing excretion in faeces, which leads to a reduction in plasma cholesterol concentrations [38,39].

Table 4 shows the results for total proteins, albumin, aspartate aminotransferase (AST), and alanine aminotransferase (ALT) in the plasma of the animals from control and treated groups.

There were no significant differences between the groups regarding total protein and albumin values. However, the AST and ALT values, two clinical markers of liver injury, were significantly higher in the PCG than in the NCG group. This result indicates the occurrence of liver damage caused by the deposition of excess liver lipids (hepatic steatosis) in PCG animals. Liao, Lin, & Kuo [34] observed a significant increase in plasma concentrations of AST and ALT in hamsters with elevated cholesterol through diet compared to normal animals.

In our study, animals fed with the whole cooked mung bean diet and animals fed with the germinated mung bean diet had significantly lower plasma concentrations of AST and ALT than PCG, despite having ingested the same amounts of saturated fat and cholesterol. This result indicates a protective effect of mung beans against liver injury caused by the hepatic deposition of excess lipids.

Another study of mung beans showed a reduction in the concentrations of AST and ALT in animals treated with aqueous extract of fermented and germinated mung beans, rich in antioxidant compounds, in a model of alcohol-induced hepatic steatosis, compared to untreated animals [40]. Similarly, Bellassoued et al. [41] found lower concentrations of these enzymes in rats fed hypercholesterolemic diets when they were treated with fermented green tea (konbucha) as compared with untreated animals. These authors attributed this protective effect to the antioxidant potential of fermented green tea.

**Figure 2:**
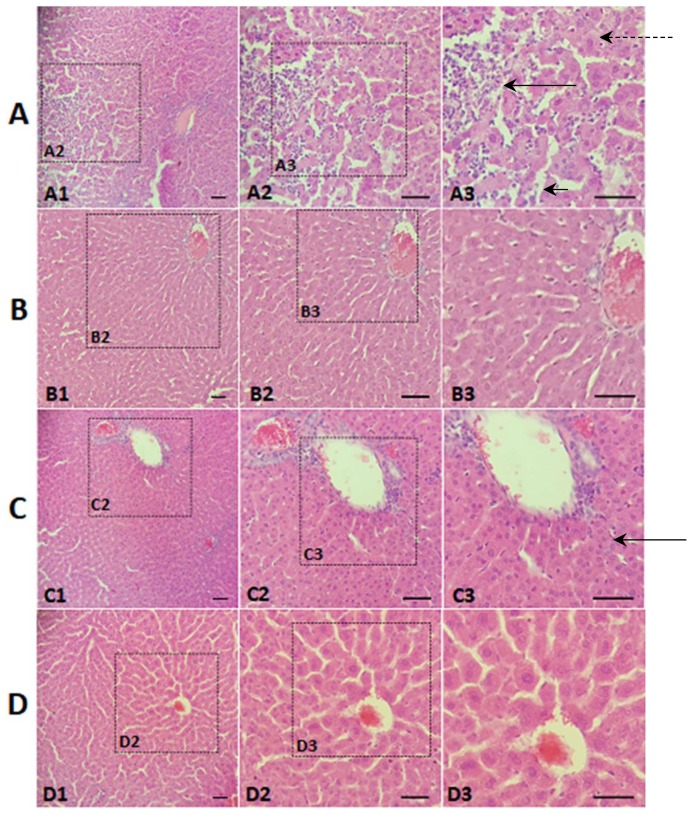
Photomicrographs of histological sections of hepatic tissue, stained with haematoxylin and eosin. Zoom: 10×, 20×, 40×: 200 μm, 100 μm, 50 μm. (**A**) Positive control group (PCG); (**B**) Negative control group (NCG); (**C**) Mung bean flour group (WMBG); (**D**) Germinated mung bean flour group (GMBG). Long black arrow: Inflammatory infiltrate, Short black arrow: Necrotic cell, Long black dotted arrow: Steatosis (microvesicular). Results of the semi-quantitative analysis of the histopathological findings: NCG: 1+, PCG: 4+, WMBG: 2.8+, GMBG: 2.2+. (Consider: 1+ = no change; 2+ = minor changes; 3+ = moderate changes; 4+ = severe changes).

The liver-protective effect of the mung bean is better observed in the examination of histological slides (Figure 2). Figure 2A shows the normal hepatocytes, preservation of hepatic parenchyma, and vascular architecture characterising normal, healthy hepatic tissue. The main findings on the histological laminae of PCG animals (Figure 2B) were: accumulation of lipid droplets in the hepatocytes (micro-vesicular steatosis), presence of areas with severe congestion and severe inflammatory infiltrate, large numbers of macrophages and Kupffer cells, vacuolar degeneration, loss of tissue architecture and hepatocyte boundaries, extensive necrotic areas, and characteristics compatible with hepatic steatosis [42].

Liver sections from WMBG animals (Figure 2C) displayed marked changes in the nucleus/cytoplasm relationship, namely, the abundance of cytoplasm in hepatocytes, moderate vascular congestion, the presence of macrophages, and the absence of steatosis. On the other hand, liver sections from GMBG (Figure 2D) animals showed a discrete increase in the nucleus/cytoplasm relationship, mild vascular congestion, and the absence of steatosis.

Histological sections from all PCG animals were graded as 4+, indicating the presence of hepatic steatosis and severe changes in the whole group. NCG animals were graded as 1+, indicating that the whole group had normal hepatic tissue, a result already expected since these animals did not receive hypercholesterolemic agents in the diet. Grades for GMBG animals were significantly lower than WMBG animals, and both were lower than PCG animals, indicating a hepatoprotective effect of mung beans, consistent with the reduction in hepatic lipid deposition; the hepatoprotective effect of germinated beans was more marked.

Frota et al. [8] and Fontanari et al. [7] found a protective effect of cowpea and lupin on the excess deposition of lipids in hepatocytes, even with increased fat and cholesterol intake. Protein isolated from these grains also presented a liver-protective effect, although with a smaller effect than the whole grain. This indicates a liver-protective effect of the leguminous proteins and a synergistic effect of other bioactive components. Xue et al. [10] reported a hepatoprotective effect exhibited by the CP-III peptide from chickpea protein, which reduced the accumulation of hepatic lipids and promoted an increase in lipoprotein lipase (LPL) and hepatic lipase (LH) activities.

Bioactive compounds with antioxidant activity, which are present in legumes, act by reducing oxidative stress and free radical formation, reducing liver damage caused by excess lipid deposition in hepatocytes. Yeap et al. [23] reported the presence of phenolic compounds and other compounds with antioxidant activity in mung beans, capable of reducing hepatic steatosis and inflammation through the positive regulation of the anti-apoptotic gene *Bcl2a1a*.

Chavez-Santoscoy et al. [12] observed a liver-protective effect associated with a positive regulation of the expression of carnitine palmitoyltransferase 1 (CPT1) and a negative regulation of SREBP-1 and HMG-CoA reductase in animals receiving a hypercholesterolemic diet rich in flavonoids and saponins extracted from common beans.

The drying process in the greenhouse for WMBD and WMBF production may have caused a reduction in the content of bioactive compounds in the flours, because most of the compounds are degraded when submitted to thermal processing [43]. It is noteworthy that studies using unprocessed germinated mung beans could have a potentially greater effect.

## 4. Conclusions

Both whole cooked mung beans and germinated mung beans promoted a reduction of total cholesterol and non-HDL cholesterol, even with the ingestion of excess fatty acids and cholesterol. This effect is partly attributed to the increase in the faecal excretion of cholesterol which occurred throughout the study in the animals treated with whole cooked mung beans and germinated mung beans. In addition, both cooked mung beans and sprouted mung beans in the diet were associated with reduced plasma levels of indicators of liver damage (aspartate aminotransferase and alanine aminotransferase). Whole cooked mung beans in the diet protected against the deposition of hepatic lipids. However, diets containing germinated mung beans showed more pronounced effects, demonstrating the absence of steatosis and inflammatory infiltrates, and better vascularisation of hepatic tissue. Therefore, the germination of mung beans appeared to enhance their liver-protective properties.

## Figures and Tables

**Table 1:** Centesimal composition of mung bean (*Vigna radiata* L.) cooked (WMBF) and germinated (GMBF) flours, on a dry matter basis.

Component (%)	WMBF	GMBF
Moisture	7.4 ± 0.6 ^b^	7.0 ± 0.3 ^a^
Ash	3.4 ± 0.1 ^a^	4.1 ± 0.1 ^b^
Lipid	1.3 ± 0.1 ^b^	0.6 ± 0.1 ^a^
Proteín	23.4 ± 0.2 ^a^	26.2 ± 0.4 ^b^
Carbohydrate ^A^	72.0 ± 0.2 ^b^	69.0 ± 0.3 ^a^

Mean ± SD. ^A^ Calculated by difference, including fibre. Mean values in lines with different letter superscripts, within each component, are statistically different at *p* < 0.05 (Student’s *t*-test).

**Table 2:** Planned and determined centesimal composition of the control diets and experimental diets.

**Planned Composition of the Diets (g/kg Dry Matter)**				
Ingredients (g/kg)	CD ^A^	CHD	WMBD	GMBD
Caseín ^B^	-	221	-	-
Whole mung bean	-	-	855	-
Germinated mung bean	-	-	-	769
L-methionine	-	-	3	3
Sucrose	-	50	50	50
Corn starch	-	427.5	-	-
Microcrystalline cellulose	-	100	60	-
Soy oil	-	20	8	14
Coconut oil	-	130	130	130
Cholesterol	-	1	1	1
Choline bitartrate	-	2.5	2.5	2.5
Mineral mix AIN 93 ^C^	-	35	35	35
Vitamin mix AIN 93 ^C^	-	10	10	10
BHT	-	0.024	0.024	0.024
**Determined Centesimal Composition of the Diets (g/100 g Dry Matter)**				
Moisture	10.2 ± 0.1 ^c^	6.8 ± 0.2 ^a^	7.9 ± 0.2 ^b^	7.3 ± 0.2 ^a^
Ash	7.8 ± 0.1 ^d^	4.8 ± 0.2 ^b^	5.4 ± 0.1 ^c^	4.1 ± 0.6 ^a^
Lipid	3.3 ± 0.1 ^c^	14.5 ± 0.1 ^b^	12.7 ± 0.1 ^a,b^	11.8 ± 0.2 ^a^
Proteín (N × 6.25)	20.9 ± 0.4 ^a^	19.1 ± 1.0 ^a^	20.3 ± 0.2 ^a^	18.9 ± 0.2 ^a^
Carbohydrate ^D^	68.2 ± 0.3 ^c^	61.3 ± 0.8 ^a^	61.5 ± 0.6 ^a^	65.5 ± 0.4 ^b^
TEV (KJ/g)	16.36	19.0	18.6	18.7

Commercial diet (CD); Hypercholesterolemic Casein Diet (CHD); Whole Mung Bean Diet (WCMBD) Germinated Mung Bean Diet (GMBD). Mean ± SD. Mean values in lines with different letter superscripts, within each component, are statistically different at *p* < 0.05 (ANOVA followed by Tukey’s test). ^A^ Commercial diet Labina^®^, manufacturer Purina^®^. ^B^ 90.4% protein. ^C^ In accordance with AIN-93 recommendations for growing rodents (Reeves et al., 1993). ^D^ Carbohydrate calculated by difference, including dietary fibre. TEV: Total Energy Value.

**Table 3:** Lipid profile and faecal cholesterol excretion in hamsters at the end of the experiment in the negative control (NCG) and positive control (PCG) groups and experimental groups fed with diets containing whole (cooked) mung bean flour (WMBG) and germinated mung bean flour (GMBG).

Lipid Parameters	NCG	PCG	WMBG	GMBG
Total cholesterol (mg/dL)	93.1 ± 4.1 ^a^	191.0 ± 12.6 ^c^	151.4 ± 5.3 ^b^	145.3 ± 6.4 ^b^
Triacylglycerols (mg/dL)	31.9 ± 5.7 ^a^	88.8 ± 4.5 ^b^	68.7 ± 7.3 ^b^	78.0 ± 9.3 ^b^
HDL-cholesterol (mg/dL)	24.7 ± 1.2 ^a^	44.8 ± 3.8 ^b^	41.0 ± 3.6 ^b^	38.4 ± 1.1 ^b^
Non-HDL cholesterol (mg/dL) ^A^	68.4 ± 3.6 ^a^	146.2 ± 12.5 ^c^	110.4 ± 5.5 ^b^	106.8 ± 5.7 ^b^
Faecal cholesterol (μmol/d/BW)	2.9 ± 0.4 ^a^	5.8 ± 0.7 ^b^	11.1 ± 0.3 ^c^	11.3 ± 0.4 ^c^

Mean ± SE. Mean values in lines with different letter superscripts are statistically different at *p* < 0.05 (ANOVA followed by Tukey’s test). ^A^ Non-HDL cholesterol = Total cholesterol − HDL cholesterol.

**Table 4:** Total proteins, albumin, aspartate aminotransferase (AST), alanine aminotransferase (ALT) in negative control (NCG), positive control (PCG), and whole (WMBG) and germinated (GMBG) mung bean groups.

Analysis	NCG	PCG	WMBG	GMBG
Total protein ^A^ (g/dL)	5.6 ± 0.1	5.92 ± 0.1	5.77 ± 0.1	5.85 ± 0.2
Albumin ^A^ (g/dL)	2.1 ± 0.5	2.23 ± 0.2	2.15 ± 0.5	2.22 ± 0.5
AST ^B^ (U/L)	61.4 ± 5.7 *	110.16 ± 6.9	74.90 ± 8.0 *	56.75 ± 7.3 *
ALT ^B^ (U/L)	73.7 ± 6.9 *	105.16 ± 21.5	75.70 ± 4.1 *	65.37 ± 8.6 *

Mean ± SE. ^A^ No significant difference *p* < 0.05 (ANOVA followed by Tukey’s test). ^B,^* significant difference *p* < 0.05 compared with PCG (Kruskal-Wallis test followed by Mann-Whitney test).
